# A systematic review and meta-analysis of high-intensity interval training for mental health and executive function in university students

**DOI:** 10.3389/fpsyg.2026.1891095

**Published:** 2026-07-24

**Authors:** Xiaoning Zhu, Zao Ni, Menglong Lian, Hang Yin

**Affiliations:** 1 Hebi Institute of Engineering and Technology, Henan Polytechnic University, Hebi, Henan, China; 2 Sichuan Film and Television University, Chengdu, Sichuan, China; 3 Zhengzhou Vocational College of Automobile Engineering, Zhengzhou, China; 4 Department of Sports Studies, Faculty of Educational Studies, Universiti Putra Malaysia, Serdang, Malaysia

**Keywords:** executive function, high-intensity interval training, mental health, meta-analysis, university students

## Abstract

**Introduction:**

University students commonly experience mental health challenges and cognitive burdens associated with academic stress and sedentary lifestyles. High-intensity interval training has been proposed as a time-efficient strategy to improve psychological and cognitive outcomes; however, evidence in university populations remains inconsistent.

**Methods:**

Web of Science, Scopus, PubMed, SportDiscus, and MEDLINE were searched from database inception to December 2024, with an updated search performed in April 2026. Randomized controlled trials investigating HIIT interventions in university students were included. Multilevel random-effects meta-analyses, subgroup analyses, exploratory moderator analyses, and sensitivity analyses were conducted.

**Results:**

Seventeen studies met the inclusion criteria. HIIT was associated with significant reductions in mental ill-health outcomes (SMD = −0.732, 95% CI: −1.236 to −0.229, *p* < 0.05; I^2^ = 84.34%). Domain-specific analyses showed significant reductions in stress and depression, whereas no significant effect was observed for anxiety. Interventions lasting ≤4 weeks showed greater effects than longer interventions. For executive function, the overall pooled effect was not statistically significant (SMD = 0.400, 95% CI: −0.006 to 0.807, *p* = 0.054; I^2^ = 70.18%). Significant improvements were identified for inhibition and shifting, but not for working memory. The certainty of evidence was very low.

**Discussion:**

Current evidence suggests that HIIT may reduce some mental ill-health outcomes among university students and may benefit selected executive function domains, but evidence for overall executive function remains inconclusive.

**Systematic review registration:**

https://www.crd.york.ac.uk/PROSPERO/view/CRD42025632509, Identifier CRD42025632509.

## Introduction

The rising prevalence of mental health challenges and sedentary lifestyles among university students is a global concern. Transitioning from adolescence to early adulthood, university students face a unique set of pressures, including academic responsibilities, social adjustments, and lifestyle changes, which can contribute to stress, anxiety, and physical inactivity (World Health Organization, 2022; Liu M. et al., 2024). These factors not only impact their immediate wellbeing but may also negatively influence executive function and academic performance (Salas-Gomez et al., 2020; del-Valle et al., 2024). In addition, prolonged sedentary behavior and insufficient physical activity may further impair cognitive health and increase the risk of long-term physical and psychological disorders, such as obesity, cardiovascular disease, and mental health problems (Cahuas et al., 2020; Zhai et al., 2021; de Santana et al., 2023). Effective interventions that address both mental wellbeing and executive functioning are therefore critical for promoting holistic health among university students.

As a widely recognized exercise method, high-intensity interval training (HIIT) has shown benefits for improving both physical and psychological outcomes. Characterized by alternating short bursts of high-intensity activity with periods of rest or low-intensity exercise, HIIT is known for its time efficiency and adaptability, making it particularly suitable for university students with busy schedules (Yin et al., 2025). Previous research has demonstrated the utility of HIIT for enhancing physical fitness, reducing body fat, and improving cardiovascular health in diverse populations, including children, adolescents, and adults (Costigan et al., 2015; Eddolls et al., 2017; Go et al., 2021; Lu et al., 2023). Additionally, growing evidence suggests that HIIT may positively influence mental health, reducing stress, anxiety, and depressive symptoms (i.e., internalizing problems), while also enhancing cognitive performance such as attention and executive functions (i.e., working memory, task switching, and inhibition) (Leahy et al., 2020; Martland et al., 2020, 2022; Liu K. et al., 2024).

Previous studies have demonstrated that HIIT may improve physical fitness, mental health, and cognitive performance across different populations, including adolescents, older adults, and the general population (Alves et al., 2021; Bauer et al., 2022; Poon et al., 2023). However, these reviews have rarely focused specifically on university students, despite this population experiencing unique academic, psychological, and lifestyle-related stressors (Daniyarova et al., 2023; Renn and Reason, 2021). Furthermore, the existing evidence among university students remains fragmented and methodologically heterogeneous. Previous studies have used diverse HIIT protocols, intervention durations, and outcome measures, particularly for executive function assessments, making direct comparisons difficult (Walsh et al., 2018; Martínez-Díaz and Carrasco, 2021; Philippot et al., 2022; Shah et al., 2022; Wang Y. et al., 2023). Several studies have also been limited by small sample sizes and varying methodological quality. As a result, the effectiveness of HIIT for improving mental health and executive function among university students remains unclear.

Given the high prevalence of psychological distress and cognitive burden among university students worldwide (Li et al., 2022; Tan et al., 2023), a comprehensive synthesis of the available evidence is warranted. Therefore, the present systematic review and meta-analysis aimed to evaluate the effects of HIIT on both mental health and executive function outcomes among university students, while also exploring potential sources of heterogeneity through subgroup analyses, exploratory moderator analyses, and sensitivity analyses.

## Method

Our study adhered to PRISMA guidelines (Page et al., 2021) and was registered in PROSPERO (CRD42025632509).

### Search strategy

We conducted a comprehensive literature search using the electronic databases Web of Science, Scopus, PubMed, SportDiscus, and MEDLINE, with the search period ending in December 2024. An updated literature search was conducted in April 2026 to ensure that the most recent studies were included and to identify any additional eligible articles published after the initial search. The same search strategy and inclusion criteria were applied in the updated search. Our search strategy employed Boolean operators, combining terms with AND and OR (e.g., HIIT AND (Cognitive OR Mental) AND University). The specific search terms and combinations used are shown in Supplementary Table S1. Moreover, reference lists of the included articles were screened to check for any additional relevant studies potentially overlooked in the initial database search, ensuring a comprehensive review of the literature.

### Eligibility criteria

Our review utilized the PICOS framework (as shown in Table 1), which encompasses five key components: Population (P), Intervention (I), Comparators (C), Main Outcomes (O), and Study Design (S). The screening process was conducted independently by XNZ and MLL, who evaluated the studies based on the predefined inclusion criteria. This evaluation included a review of titles, abstracts, and full-text articles to determine their eligibility for inclusion. In cases where discrepancies arose, a third author (ZN) was consulted to resolve disagreements and ensure consensus.

**Table 1:** Eligibility criteria according to PICOS.

Items	Inclusion criteria	Exclusion criteria
Population	Individuals are currently enrolled part-time or full-time at a university or college	Studies involving other populations (e.g., children, adolescents, older adults, clinical populations)
Intervention	Meet the definition of HIIT (Paul and Martin, 2019), lasting for at least 2 weeks, and chronic studies	Studies that focus on other forms of physical activity or exercise that do not meet the HIIT criteria
Comparator	Studies with a control group (e.g., non-exercise, low/moderate-intensity exercise)	Studies without a clear comparison group or pre-post assessment
Outcome	Psychological or cognitive-related outcomes	Studies focusing solely on physiological, metabolic, or physical performance outcomes without psychological or cognitive measures
Study design	Randomized controlled trials	Non-randomized controlled trials, Observational studies, qualitative studies, case reports, cross-sectional studies
Additional criteria	Published in English, peer-reviewed, and full-text accessible	Conference abstracts, reviews, commentaries, editorials, systematic reviews, dissertations, debates, posters, proposed studies, and grey literature

### Data extraction

The data extraction process was conducted independently by XNZ and MLL, with any disagreements resolved by ZN. The extracted data included key information such as the author, year of publication, study location, sample size, gender ratio, and participant age. Additionally, intervention details were recorded, including the HIIT approach used, the duration of the protocol, rest intervals, total intervention duration, and intervention frequency. For the primary meta-analysis, all comparator types (including inactive controls, health education, and low/moderate-intensity exercise) were pooled together. Furthermore, data on psychological and cognitive-related outcomes were systematically collected.

### Assessment of risk bias

Risk of bias was assessed using the Cochrane Risk of Bias 2 tool (RoB 2), which is specifically designed for randomized controlled trials (Sterne et al., 2019). Two reviewers independently evaluated each included study across five domains: randomization process, deviations from intended interventions, missing outcome data, measurement of outcomes, and selection of the reported result. Each domain, as well as the overall risk of bias, was judged as “low risk,” “some concerns,” or “high risk” according to RoB 2 guidance. Disagreements were resolved through discussion with a third reviewer, and the risk-of-bias judgements were considered when interpreting the findings and grading the certainty of evidence.

### Statistical analysis and meta-analysis

All statistical analyses were performed in RStudio using the metafor package. Effect sizes were calculated as Cohen’s d to quantify the standardized between-group difference between the HIIT and control conditions. For the primary analyses, effect sizes were calculated using post-intervention means and standard deviations. When post-intervention data were unavailable, change-score data were extracted where reported, and adjusted effects were used only when unadjusted post-intervention or change-score data were unavailable and could be converted to a standardized metric. Baseline differences were not directly adjusted when post-intervention means were used; however, baseline comparability was considered in the risk-of-bias assessment, and adjusted estimates were preferentially extracted when substantial baseline imbalance was reported with suitable adjusted results. Effect sizes of approximately 0.2, 0.5, and 0.8 were interpreted as small, moderate, and large, respectively (Cohen, 1988).

For mental health outcomes, the direction of effect sizes was retained according to the original scoring of each measure. Consequently, negative effect sizes indicated beneficial reductions in adverse outcomes such as stress, anxiety, depression, or burnout in the intervention group compared with the control group. For executive function outcomes, effect sizes were standardized so that positive values consistently reflected improved executive function performance following the intervention. Therefore, for measures in which lower scores represented better performance (e.g., reaction time and error rates), effect sizes were multiplied by −1 before analysis. Measures in which higher scores indicated better performance (e.g., accuracy, correct responses, and digit span scores) were retained without transformation.

Because several studies contributed more than one eligible outcome, a multilevel random-effects meta-analysis was used to account for the statistical dependence among effect sizes from the same study. The model included effect sizes nested within studies, with random effects specified at both the study level and the within-study effect-size level. This structure allowed the analysis to retain multiple relevant outcomes without selecting a single effect size from each study or treating dependent effects as independent (Cheung, 2014). For studies that included more than one eligible control group, the non-exercise or usual-care control group was selected for the primary analysis to maintain comparability across studies and avoid double-counting participants. Adverse psychological outcomes, including stress, anxiety, depression, obsessive-compulsive symptoms, and burnout, were grouped under the broad category of mental ill-health to provide an overall estimate of the effect of HIIT on negative psychological outcomes. This approach was chosen because the number of studies available for each individual construct was limited and because these outcomes represent related, although clinically distinct, dimensions of psychological distress. To avoid overinterpreting this composite outcome, domain-specific subgroup analyses were conducted for stress, depression, and anxiety where sufficient data were available, and these analyses were emphasized in the interpretation of the findings. The I^2^ statistic was used to evaluate heterogeneity, with values below 25%, between 25 and 50%, and above 75% interpreted as low, moderate, and high heterogeneity, respectively (Higgins, 2011).

### Certainty of evidence

The certainty of evidence for the major outcomes was determined using the GRADE approach (Guyatt et al., 2011). Several domains were considered during the assessment process, including risk of bias, inconsistency, indirectness, and imprecision. Based on the overall evaluation, the certainty of evidence was rated as high, moderate, low, or very low, thereby supporting interpretation of the strength and stability of the meta-analytic findings.

## Results

### Characteristics of included studies

**Figure 1:**
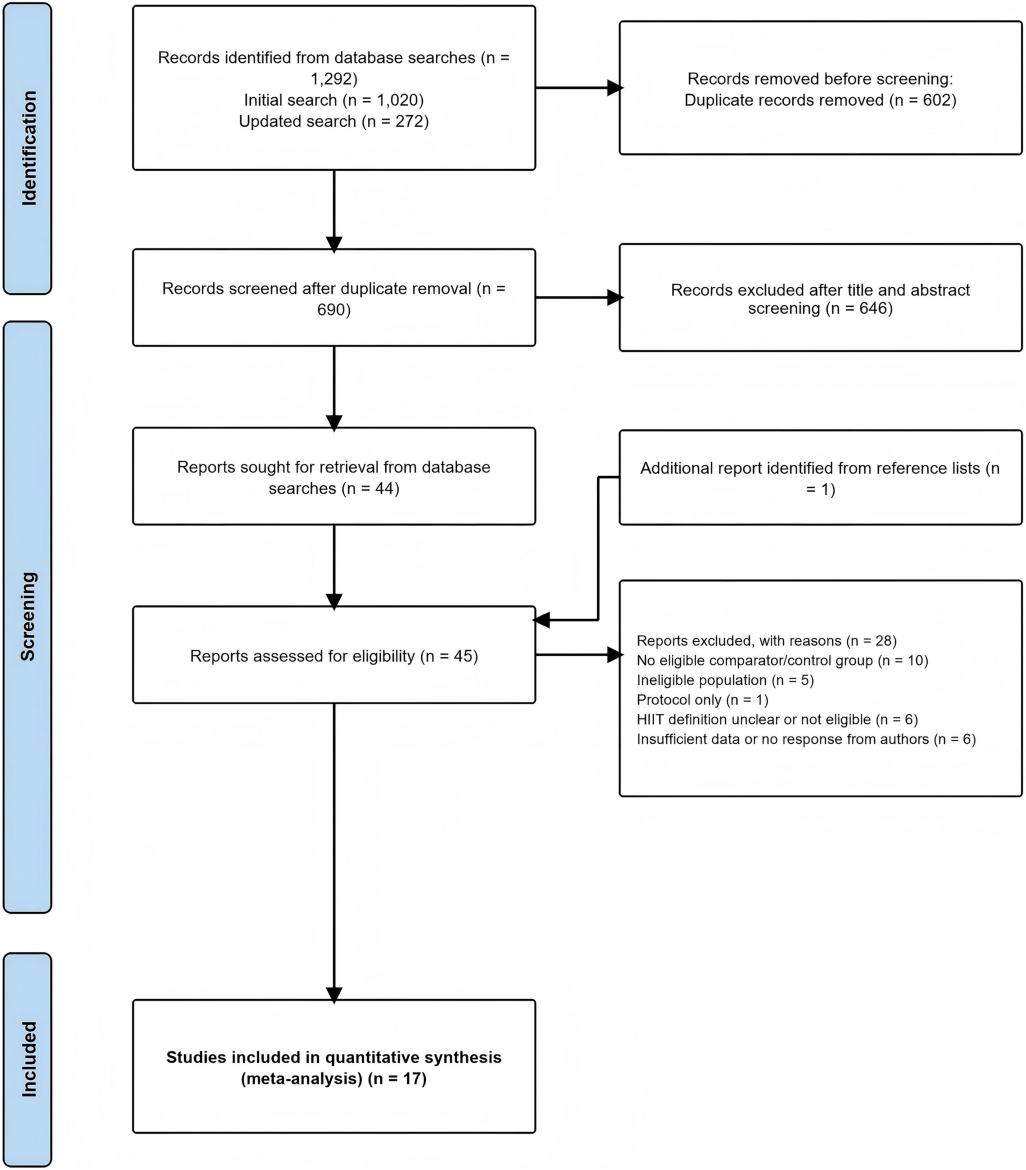
Flow chart.

A total of 1,292 articles were identified through database searches. After removing duplicates (*n* = 602), 690 articles remained for title and abstract screening, followed by full-text review (*n* = 45). Ultimately, 17 studies met the inclusion criteria for analysis (a detailed flowchart is presented in Figure 1). Table 2 presents the main characteristics of the included studies. In summary, all 17 studies employed an RCT design. Sample sizes ranged from 24 to 93 participants, including both males and females, with samples drawn from nine different countries: China (Zhang et al., 2021; Liu et al., 2022; Guo et al., 2023; Wang X. et al., 2023; Wang Y. et al., 2023; Chang et al., 2024; Yin et al., 2024; Fu et al., 2025), South Africa (Nduduzo et al., 2023), India (Kaur and Rizvi, 2024; Thenmozhi et al., 2025), Belgium (Philippot et al., 2022), Turkey (Yalman et al., 2021), Canada (Lucibello et al., 2020), Australia (Eather et al., 2019), Germany (Wallenwein et al., 2025), and Spain (Jimenez-Roldán et al., 2025). Regarding the forms of HIIT, 4 studies utilized bike-based exercises (Lucibello et al., 2020; Yalman et al., 2021; Nduduzo et al., 2023; Wang X. et al., 2023), 10 studies employed combined motion exercises (e.g., jumping jacks, squats, deadlifts, push presses, burpees) (Eather et al., 2019; Zhang et al., 2021; Philippot et al., 2022; Guo et al., 2023; Wang Y. et al., 2023; Kaur and Rizvi, 2024; Fu et al., 2025; Jimenez-Roldán et al., 2025; Thenmozhi et al., 2025), two studies used running exercises (Liu et al., 2022; Chang et al., 2024), and one study used stair-climbing exercises (Yin et al., 2024). Intervention durations ranged from 2 to 12 weeks, with only two studies implementing a frequency of more than 4 sessions per week (Kaur and Rizvi, 2024; Fu et al., 2025).

**Table 2:** Summary of included studies.

Study/Country/study design	Sample size/age/gender	Intervention protocol	Mental and cognitive related outcomes	Mental and cognitive related results
Wang X. et al. (2023) China RCT	81/22.45 ± 2.49/63% male	HG: All-out bike exercise (work: rest = 1:1) CG: Keep normal living conditions 2 times per week in 6 weeks	Executive Function	Inhibition: *d* = 0.21, +2.16% Shifting: *d* = 0.21, +2.11% Working memory (ms): *d* = 0.19, +3.51%
Nduduzo et al. (2023) South Africa RCT	40/ NR/ 100% male	HG: Bike exercise (85% HRmax for 20 min) (work: rest = 2:3) CG: No normal physical exercise 3 times per week in 8 weeks	Quality of life (Psychological health)	Psychological health: *d* = 1.25, +25.56%
Kaur and Rizvi (2024) India RCT	60/21.07 ± 1.48/40% male/	HG: Combined motion exercise (6–9 RPE) (work: rest = 2:1) CG: received health education 5 times per week in 6 weeks	Mental outcome (burnout)	Occupational exhaustion: *d* = −2.7, −32.23% Depersonalization: *d* = −3.47, −40.65% Personal accomplishment: *d* = 4.85, +23.08% GHQ: *d* = −0.99, −24.45%
Yin et al. (2024) China RCT	42/21.94 ± 2.61/43% male	HG: All-out stair climbing CG: Keep normal living conditions 3 times per week in 6 weeks	Mental outcome	Stress: *d* = −0.41, −6.30%
Philippot et al. (2022) Belgium RCT	28/20.79 ± 1.70/11% male	HG: Combined motion exercise (above 80% HRmax) (work: rest = 1:1) CG: Keep normal living conditions 3 times per week in 4 weeks	Psychological symptoms	Depression: *d* = −0.76, −45.45% Anxiety: *d* = −0.41, −26.32% Stress: *d* = −0.53, −25.49%
Wang Y. et al. (2023) China RCT	60/19.75 ± 1.55/26% male	HG: Remote coaching HIIT CG: Combined exercise (work: rest = NR) 3 times per week in 8 weeks	Psychological health	Obsessive-compulsive: *d* = −0.15, −3.76% Depression: *d* = 0.32, +4.5% Anxiety: *d* = 0.57, +10.19% Total PH d = 0.17, +2.27%
Yalman et al. (2021) Turkey RCT	36/20.83 ± 0.97/42% male	HG: 90%HRmax bike exercise (work: rest = 1:3) CG: 60–70% HRmax 3 times per week in 4 weeks	Psychological symptoms	Stress: *d* = −0.64, −15.85%
Eather et al. (2019) Australia RCT	53/20.38 ± 1.88/34% male	HG:85% or above HRmax combined motion exercise (work: rest = 1:1) CG: Keep normal living conditions 3 times per week in 8 weeks	Executive function Psychological outcomes	Stress: *d* = −0.64, −19.85% Anxiety: *d* = 0.09, 1.34% Shifting: *d* = 0.27, +8.52%
Lucibello et al. (2020) Canada RCT	46/19.9 ± 2.2/37% male	HG: 90-95%HRmax bike exercise (work: rest = 1:1) CG: Keep normal living conditions 3 times per week in 9 weeks	Mental outcomes	Anxiety: *d* = −0.19, −23.33% Depression: d = 0.20, 2.12%
Guo et al. (2023) China RCT	48/20.42 ± 1.75/100% female	HG:85%HRmax combined motion exercise (work: rest = more than 1:1) CG: Keep normal living conditions 3 times per week in 4 weeks	Executive function Psychological outcomes	Inhibition: *d* = 1.11, +18.69% Depression: *d* = −2.04, −14.51%
Chang et al. (2024) China RCT	24/20.84 ± 1.14/ NR	HG: 90%MAS running (work: rest = more than 1:1.25) CG: 90%MAS running 3 times per week in 12 weeks	Mental symptoms	Stress: *d* = −0.14, −4.92% Anxiety: *d* = −1.25, − 26.42% Depression: *d* = −1.98, −50.79%
Liu et al. (2022) China RCT	93/25.26 ± 2.21/43% male	HG:100%VO_2_max Running (work: rest = more than 1:1) CG: 1 h of health education twice a week 3 times per week in 12 weeks	Mental symptoms	Wellbeing: *d* = 0.45, 17.20%
Zhang et al. (2021) China RCT	62/22.83 ± 2.20/100% female	HG: 80% above HRmax combined motion exercise (work: rest = more than 1:1) CG: Health education course 3 times per week in 6 weeks	Mental outcomes	Anxiety: *d* = −0.12, −4.38% Stress: *d* = −0.14, −6.89%
Fu et al. (2025) China RCT	35/19.25 ± 1.19/100% female	HG: 90% VO_2_ above combined motion exercise (work: rest = more than 2:1) CG: Standard health education 5 times per week in 4 weeks	Mental health	Depression: *d* = −0.82, −44.83% Anxiety: *d* = −0.74, −48.15%
Thenmozhi et al. (2025) India RCT	40/18–23/ NR	HG: combined motion exercise CG: regular daily routines (work: rest = NR) 3 times per week in 12 weeks	Mental outcomes	Stress: *d* = −3.02, −15.54%
Wallenwein et al. (2025) Germany RCT	58/22.59 ± 3.05 50% female	HG: Jump training CG: Regular schedule (work: rest = dynamic changes) 3 times per week in 8 weeks	Executive function	Inhibition: *d* = 0.17, +8.87% Shifting: *d* = 0.15, +4.24% Working memory: *d* = −0.73, −5.56%
Jimenez-Roldán et al. (2025) Spain RCT	52/22 ± 1.1/100% female	HG: RPE8-10 HIIT CG: Usual daily routines (work: rest = 1:1) 3 times per week in 12 weeks	Executive function	Inhibition: *d* = 0.49, +13.62% Shifting: *d* = 0.99, +24.56% Working memory: *d* = 0.76, +13.73%

RCT, Randomized controlled trial; HG, HIIT group; CG, Control group; RPE, Rating of Perceived Exertion; GHQ, General health questionnaire; ART, Aerobic and resistance training; PH, Psychological health; MAS, Maximal Aerobic Speed.

### Assessment of risk of bias

**Figure 2:**
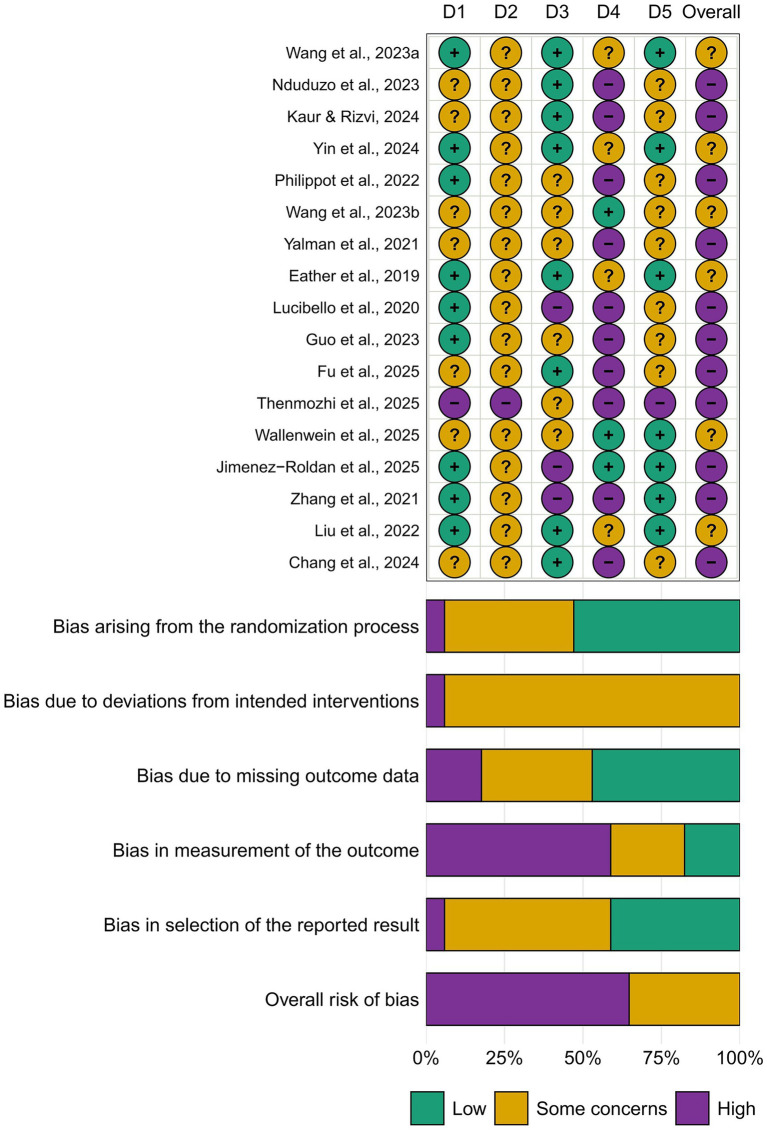
Risk of bias.

The RoB 2 assessment is presented in Figure 2. None of the included studies was judged to have a low overall risk of bias; six studies were rated as having “some concerns” and eleven as having a high overall risk of bias. The most frequent sources of bias were deviations from intended interventions and outcome measurement, with most studies showing some concerns in Domain 2 and a high risk of bias in Domain 4. Risk of bias arising from the randomization process, missing outcome data, and selection of the reported result was more variable across studies, with several trials rated as low risk in these domains. Overall, the risk-of-bias assessment indicated important methodological limitations across the included RCTs, which should be considered when interpreting the pooled estimates and grading the certainty of evidence.

### Certainty of evidence

The GRADE assessment indicated that the certainty of evidence was very low for both mental ill-health and executive function outcomes (Table 3). For mental ill-health, the evidence was downgraded because of serious risk of bias, substantial inconsistency, and imprecision related to the limited sample size and wide confidence interval. Although the pooled effect favored HIIT, heterogeneity was considerable (I^2^ = 84.34%), and several included studies were judged to have high overall risk of bias. For executive function, the evidence was also rated as very low because of serious risk of bias, substantial heterogeneity (I^2^ = 70.18%), and imprecision, as the overall pooled estimate crossed the line of no effect and did not reach statistical significance. Publication bias could not be formally assessed because of the limited number of studies and the dependent structure of effect sizes; therefore, small-study effects could not be excluded.

**Table 3:** GRADE result.

Outcomes	Grade assessment					Effect size (95%CI)	Certainty of evidence
	Risk of bias	Inconsistency	Indirectness	Imprecision	Others		
MIH	Serious	Serious	Not serious	Serious	None	Cohen’s d = −0.72, 95%CI: [−1.25, −0.20], *p* < 0.05	Very low
EF	Serious	Serious	Not serious	Serious	None	Cohen’s d = 0.40, 95%CI: [−0.01, 0.81], *p* = 0.054	Very low

MIH, Mental ill-health; EF, Executive function; Risk of bias was rated as serious based on the RoB 2 assessment. Inconsistency was rated as serious because substantial heterogeneity was observed across outcomes. Imprecision was rated as serious because of limited sample sizes and wide confidence intervals. Publication bias was not formally assessed because of the limited number of studies and dependent effect sizes; therefore, potential small-study effects could not be excluded.

### Effects of HIIT on mental ill-health

The multilevel meta-analysis for mental ill-health outcomes (Table 4) demonstrated a significant overall effect favoring HIIT (SMD = −0.732, 95% CI: −1.236 to −0.229, *p* < 0.05), with substantial heterogeneity (I^2^ = 84.34%). Because the composite mental ill-health outcome included clinically distinct constructs, the overall pooled estimate was interpreted alongside domain-specific subgroup analyses. Subgroup analyses (Supplementary Figure S1) showed that HIIT significantly reduced stress (SMD = −0.756, 95% CI: −1.433 to −0.079, *p* < 0.01) and depression (SMD = −0.850, 95% CI: −1.640 to −0.060, p < 0.05). However, no significant effect was observed for anxiety (SMD = −0.240, 95% CI: −0.643 to 0.164, *p* = 0.244). Considerable heterogeneity was identified across the subgroup analyses, with I^2^ values ranging from 63.35 to 86.33%.

**Table 4:** Meta-analysis of the effect of HIIT on mental ill-health and executive function.

Outcomes	Effect size and precision						Heterogeneity			
	*k*	*N*	Estimate	95%CI (L)	95%CI (U)	*p*	*QE*	*df (Q)*	*Q_P*	*I^2^*
Mental ill-health	21	848	−0.732	−1.236	−0.229	*	96.129	20	***	84.34%
Subgroup										
Stress	7	272	−0.756	−1.433	−0.079	**	29.337	6	***	83.64%
Depression	6	230	−0.850	−1.640	−0.060	*	40.373	5	***	86.33%
Anxiety	7	297	−0.240	−0.643	0.164	0.244	16.594	6	*	63.35%
Exploratory moderator analysis										
Duration										
≤4 weeks	8	303	−0.756	−1.334	−0.178	**	22.736	7	**	74.16%
>4 weeks	13	545	−0.736	−1.599	0.126	0.094	68.725	12	***	90.09%
Risk of bias										
Some concerns	6	282	−0.062	−0.493	0.368	0.776	12.153	5	*	60.18%
High	15	566	−0.984	−1.628	−0.340	0.003	66.486	14	***	84.90%
Executive function	11	596	0.400	−0.006	0.807	0.054	29.569	10	***	70.18%
Subgroup										
Inhibition	4	213	0.473	0.068	0.877	*	6.503	3	0.089	53.91%
Shifting	4	218	0.387	0.023	0.751	*	5.465	3	0.141	44.237%
Working memory	3	165	0.071	−0.776	0.919	0.869	14.019	2	***	86.01%

*p < 0.05; **p < 0.01; ***p < 0.001.

Exploratory moderator analyses indicated that intervention duration and risk of bias may have contributed to variation in the pooled effects. Significant effects were found in interventions lasting ≤4 weeks (SMD = −0.756, 95% CI: −1.334 to −0.178, *p* = 0.010), whereas interventions lasting >4 weeks did not reach statistical significance (SMD = −0.736, 95% CI: −1.599 to 0.126, *p* = 0.094). When studies were grouped according to RoB 2 overall judgements, the pooled effect was not statistically significant among studies rated as having some concerns (SMD = −0.062, 95% CI: −0.493 to 0.368, *p* = 0.776), but was significant among studies rated as having high risk of bias (SMD = −0.984, 95% CI: −1.628 to −0.340, *p* = 0.003). Heterogeneity remained moderate to substantial in both subgroups (I^2^ = 60.18 and 84.90%, respectively). These findings should be interpreted cautiously because no included study was judged as having low overall risk of bias, subgroup sizes were limited, and the larger pooled effect was observed in studies at high risk of bias.

Leave-one-study-out sensitivity analyses (Supplementary Table S2) demonstrated that the pooled effects remained statistically significant after sequential omission of each study, with effect sizes ranging from −0.529 to −0.836. This suggests that the overall estimate for mental ill-health was not driven by any single study, although the finding should still be interpreted cautiously given the very low certainty of evidence, substantial heterogeneity, and risk-of-bias concerns.

### Effects of HIIT on executive function

The multilevel meta-analysis for executive function outcomes (Table 4) showed that the overall pooled effect did not reach statistical significance (SMD = 0.400, 95% CI: −0.006 to 0.807, *p* = 0.054), with substantial heterogeneity (I^2^ = 70.18%).

Subgroup analyses (Supplementary Figure S2) revealed significant positive effects of HIIT on inhibition (SMD = 0.473, 95% CI: 0.068 to 0.877, *p* < 0.05) and shifting (SMD = 0.387, 95% CI: 0.023 to 0.751, *p* < 0.05). However, no significant effect was observed for working memory (SMD = 0.071, 95% CI: −0.776 to 0.919, *p* = 0.869). Thus, evidence for executive function appeared domain-specific, with possible benefits for inhibition and shifting but not for overall executive function or working memory. Moderate heterogeneity was identified for inhibition (I^2^ = 53.91%) and shifting (I^2^ = 44.237%), whereas working memory demonstrated considerable heterogeneity (I^2^ = 86.01%).

Leave-one-study-out sensitivity analyses (Supplementary Table S2) indicated that the pooled effect estimates ranged from 0.270 to 0.549 after sequential omission of each study. Notably, omission of the study by Wallenwein et al. resulted in a statistically significant pooled effect (SMD = 0.549, 95% CI: 0.160 to 0.938, *p* = 0.006), suggesting that the overall findings may be partially influenced by this study. This sensitivity finding further supports a cautious interpretation of the overall executive function result.

### Narrative findings on other mental health outcomes

Due to the limited number of comparable effect sizes, a quantitative synthesis was not conducted for general mental health and wellbeing outcomes. Three studies reported positive effects of HIIT on overall mental health-related outcomes. One study (Nduduzo et al., 2023) observed a large improvement in psychological health (*d* = 1.25), corresponding to a 25.56% increase following the intervention. One study (Wang Y. et al., 2023) reported a small positive effect on total psychological health (*d* = 0.17), with an improvement of 2.27%. Similarly, One (Liu et al., 2022) found a moderate improvement in wellbeing (*d* = 0.45), corresponding to a 17.20% increase after HIIT participation.

## Discussion

This study aimed to synthesize the existing evidence regarding the effects of HIIT on executive function and mental health among university students. To the best of our knowledge, this is the first systematic review and meta-analysis focusing specifically on this population. The findings suggest that HIIT may be associated with improvements in some mental health and executive function-related outcomes, particularly mental ill-health, inhibition, and shifting. The multilevel meta-analysis further indicated that HIIT significantly reduced mental ill-health among university students, although the certainty of evidence was very low and heterogeneity was substantial. In contrast, the overall pooled effect on executive function did not reach statistical significance, although subgroup analyses suggested possible improvements in inhibition and shifting. Overall, these findings suggest that HIIT may represent a promising strategy for promoting mental wellbeing and selected domains of executive functioning in university students, but the results should be interpreted cautiously given the methodological limitations and uncertainty of the current evidence.

However, the certainty of evidence was rated as very low, indicating that the findings should be interpreted with caution. The design and methodological quality of the included studies varied, and several studies presented limitations that may have affected the validity of their findings. The RoB 2 assessment indicated that none of the included studies had a low overall risk of bias, with most studies judged as having either some concerns or a high risk of bias. Common issues included a lack of blinding, inadequate allocation concealment, small sample sizes, and incomplete reporting of participant adherence to intervention protocols. These methodological limitations may increase the risk of performance and detection bias, particularly for subjective outcomes such as mental health measures (Chandler et al., 2019). In addition, several outcomes reported unusually large effect sizes, which should be interpreted cautiously given the methodological limitations and relatively small samples of some included studies. This concern is further supported by the exploratory moderator analysis, in which the pooled mental ill-health effect was significant among studies judged as having a high risk of bias but not among those rated as having some concerns. Poor adherence to intervention protocols may also dilute the observed effects of HIIT, potentially leading to underestimation or overestimation of intervention efficacy. Therefore, future studies should adopt more rigorous methodological designs and provide transparent reporting in accordance with established guidelines such as CONSORT (Schulz et al., 2010) to improve the reliability and reproducibility of findings.

The intervention strategies employed in the included studies varied in duration, frequency, and exercise modality, which may have contributed to the substantial heterogeneity observed across outcomes. Intervention periods ranged from 2 weeks to 12 weeks, with most studies implementing a frequency of three sessions per week. Regarding the mode of exercise, cycling-based HIIT was the most commonly used, followed by combined motion exercises such as jumping jacks, deadlifts, squats, burpees, and mountain climbers. The other studies used running exercises and stair-climbing exercises as an intervention. While cycling-based HIIT offers precise control over intensity and workload, its reliance on equipment may limit accessibility in certain settings. In contrast, combined motion exercises, running exercises, and stair-climbing provide practical and convenient alternatives that require no specialized equipment, making them potentially suitable for university students who often face time and resource constraints. These modalities allow for greater flexibility and adaptability, enabling students to perform exercises anytime and anywhere. Prior research highlights the versatility of bodyweight HIIT, emphasizing its potential for wide-scale implementation across diverse populations and settings (Kinnafick et al., 2018; Li et al., 2023; Liang et al., 2024). Additionally, HIIT is consistently recognized as a time-efficient exercise modality that delivers significant health benefits within short durations (Gillen and Gibala, 2014). This efficiency, combined with its adaptability, makes HIIT an appealing option for university students to integrate physical activity into their daily routines, although variation in protocols should be considered when interpreting the pooled effects.

### Effects of HIIT on mental ill-health

The included studies suggested that HIIT may positively influence several aspects of mental health among university students. The multilevel meta-analysis showed a significant overall reduction in mental ill-health outcomes, with subgroup analyses further indicating significant improvements in stress and depression. However, the overall mental ill-health category combined related but clinically distinct constructs, including stress, anxiety, depression, burnout, and obsessive-compulsive symptoms. Therefore, this pooled estimate should be interpreted as a broad summary of adverse psychological outcomes rather than evidence of uniform effects across all mental health domains. These findings support the potential of HIIT as a promising strategy for alleviating psychological distress in university settings, although the very low certainty of evidence limits the strength of this conclusion. Previous evidence suggests that high-intensity exercise may stimulate the release of endorphins and other neurobiological factors associated with stress reduction and mood enhancement (Chan et al., 2019; Chen and Nakagawa, 2023). In contrast, no significant pooled effect was observed for anxiety, which may reflect differences in intervention characteristics, participant populations, or outcome measurements across studies (Huang et al., 2024). Accordingly, the domain-specific findings for stress, depression, and anxiety are likely to be more clinically informative than the overall composite estimate. In addition to the quantitative findings, several studies reported improvements in broader psychological health and wellbeing outcomes. Given the time-efficient nature of HIIT, this form of exercise may be potentially suitable for university students who often experience substantial academic demands and limited time for physical activity participation (Malagodi et al., 2025).

Exploratory moderator analyses indicated that intervention duration and risk of bias may influence the mental health effects of HIIT. Significant effects were observed in interventions lasting 4 weeks or less, whereas interventions of longer duration did not demonstrate statistically significant pooled effects. However, these findings should be interpreted cautiously because the number of studies within subgroups was limited and substantial heterogeneity remained. One possible explanation is that prolonged interventions may be associated with reduced participant adherence, motivation, or engagement over time (Peters et al., 2023). In university settings, longer intervention periods may also be more susceptible to external influences such as academic workload, examination stress, and social pressures, which could attenuate the psychological benefits of exercise participation. Therefore, the duration-related moderator finding should be considered hypothesis-generating rather than confirmatory.

Regarding risk of bias, the exploratory moderator analysis showed that the pooled effect was not statistically significant among studies rated as having some concerns in the RoB 2 assessment, whereas a significant pooled effect was observed among studies rated as having high risk of bias. This pattern suggests that the overall mental ill-health effect may be partly influenced by methodological limitations, particularly because no included study was judged as having low overall risk of bias. However, these findings should be interpreted cautiously because the number of studies within each subgroup was relatively small and substantial heterogeneity remained. In particular, several studies with a high risk of bias reported large effect sizes, which may have inflated subgroup estimates. Therefore, additional high-quality randomized controlled trials with lower risk of bias, adequate allocation procedures, transparent adherence reporting, and standardized outcome measurement are needed to further clarify the effectiveness of HIIT on mental health outcomes among university students.

### Effects of HIIT on executive function

Although the overall multilevel meta-analysis did not show a statistically significant pooled effect on executive function, subgroup analyses revealed significant improvements in inhibition and shifting following HIIT interventions, whereas no significant effect was observed for working memory. Therefore, the present findings do not support a broad conclusion that HIIT improves executive function overall; rather, they suggest possible domain-specific benefits for inhibition and shifting. These findings are partially consistent with previous research indicating that high-intensity exercise can enhance certain aspects of executive functioning through mechanisms such as increased cerebral blood flow, elevated brain-derived neurotrophic factor, enhanced catecholamine activity, and improved neuroplasticity (Stillman et al., 2016; Bhattacharya et al., 2023). In particular, inhibition and cognitive flexibility are often considered more sensitive to acute physiological arousal and exercise-induced neural activation, which may explain why these domains responded more positively to HIIT interventions (Chen and Nakagawa, 2023; Singh et al., 2025). However, given the very low certainty of evidence, the limited number of studies, and heterogeneity across executive function measures, these domain-specific findings should be interpreted cautiously.

However, the present findings also differ from some previous studies and reviews reporting broader cognitive benefits of exercise, including improvements in working memory (Martínez-Díaz et al., 2020; Liu K. et al., 2024; Yue et al., 2025). One possible explanation is the substantial heterogeneity in intervention protocols, including differences in exercise intensity, duration, frequency, and training modality (Singh et al., 2025). Furthermore, the included studies employed a wide range of executive function assessments, such as Stroop tasks, Trail Making Tests, n-back tasks, Wisconsin Card Sorting Tests, and Go/No-Go paradigms, which may capture different cognitive processes and contribute to inconsistent findings across studies. The relatively small number of studies and participants included in each executive function subgroup may have further limited the statistical power to detect significant pooled effects, particularly for working memory outcomes. In addition, the confidence interval for the overall executive function estimate crossed the line of no effect, further indicating uncertainty around the pooled estimate. These methodological and statistical limitations may explain why significant effects were observed for selected domains but not for overall executive function.

The observed improvements in inhibition and shifting may be particularly relevant within the university context. University students frequently encounter substantial academic demands, including prolonged concentration, multitasking, time management, rapid decision-making, and regulation of emotional and cognitive distractions (Pretorius and Heyns, 2026). Inhibitory control is essential for resisting distractions and maintaining attention during academic tasks, whereas cognitive flexibility (shifting) supports adaptive thinking, problem-solving, and efficient switching between different learning tasks or social demands (Ramos-Galarza et al., 2019; Lee et al., 2024). Therefore, if confirmed in future high-quality trials, improvements in these executive function domains may contribute not only to academic performance but also to students’ daily self-regulation and psychological adaptation during university life (Jacob and Parkinson, 2015). However, because the overall executive function effect was not statistically significant, these implications should be viewed as tentative and specific to inhibition and shifting rather than executive function as a whole.

### Practical implications for HIIT implementation in university settings

Given the very low certainty of evidence, the practical implications of this review should be interpreted cautiously. Nevertheless, HIIT may be a feasible and time-efficient option for university health promotion, particularly when programs are designed to be accessible, adaptable, and embedded within existing student support systems. Drawing on the conceptual model proposed by Lubans and colleagues (Lubans et al., 2022), HIIT implementation in university settings may be considered across four components: opportunity, design, delivery, and support.

#### Integration of HIIT into university programs (opportunity)

HIIT could be incorporated into existing university structures, such as physical education classes, extracurricular activities, campus recreation services, or wellness initiatives. Embedding HIIT within established programs may reduce common barriers to participation, including limited time, low motivation, and restricted access to facilities (Bull et al., 2020). However, such implementation should be accompanied by monitoring of adherence, safety, and psychological outcomes, given the current uncertainty in the evidence base.

#### Tailoring HIIT program design to enhance physical literacy (design)

HIIT programs for university students should be adaptable to different fitness levels and should allow modifications in intensity, exercise mode, and progression. Rather than promoting a single optimal HIIT protocol, current evidence supports the need for flexible designs that account for students’ baseline fitness, preferences, and academic schedules (Ryan and Deci, 2020). Programs may also include brief educational components explaining potential physical and psychological benefits, but claims regarding mental health and executive function should remain proportionate to the very low certainty of evidence.

#### Engaging and student-centered delivery of HIIT interventions (delivery)

The delivery of HIIT should prioritize clear instruction, appropriate supervision, and an engaging environment. Group-based sessions, digital platforms, or app-supported formats may help improve accessibility and adherence, particularly among students with limited time or variable schedules (Finkelstein et al., 2016; Wang et al., 2024). Qualified instructors may be important for adapting exercises, monitoring intensity, and minimizing adverse experiences, especially for inactive students or beginners.

#### Ongoing support and community engagement (support)

Sustained participation may require social and institutional support beyond the initial intervention period. Universities may consider peer-supported exercise groups, campus recreation partnerships, or links with student health and counseling services. However, future implementation should be evaluated through well-designed trials with standardized psychological and executive function outcomes, longer follow-up, and transparent reporting of adherence and adverse events.

## Limitations

Several limitations should be acknowledged. First, the number of included studies was relatively small, particularly for executive function outcomes, and several subgroup and exploratory moderator analyses were based on limited effect sizes. Second, substantial heterogeneity was observed across analyses, likely reflecting differences in HIIT protocols, participant characteristics, comparator conditions, and outcome measures. Third, the included studies had important methodological limitations; no study was judged as having low overall risk of bias using RoB 2, and the GRADE certainty of evidence was very low. Fourth, executive function assessments varied considerably across studies, limiting direct comparability. Finally, publication bias was not formally assessed because of the limited number of studies and the dependent structure of effect sizes. Nevertheless, small-study effects cannot be excluded, particularly because several studies had small samples and reported large effects, which may have inflated the pooled estimates.

## Implication and future direction

The findings have potential implications for university health promotion, but they should be interpreted cautiously given the very low certainty of evidence and the non-significant overall effect on executive function. HIIT may offer a time-efficient and flexible option for supporting student wellbeing, but it should not yet be promoted as a definitive strategy for improving both psychological and cognitive outcomes. Future studies should test optimized HIIT protocols that account for baseline fitness, gender, psychological profiles, comparator conditions, adherence, and safety. Wearable technology and gamification may help improve engagement, but these approaches should be evaluated using rigorous trial designs and standardized psychological and executive function outcomes.

## Conclusion

The current evidence suggests that HIIT may reduce mental ill-health among university students, particularly stress and depression. Evidence for overall executive function remains inconclusive, although domain-specific analyses suggest possible benefits for inhibition and shifting. Given the very low certainty of evidence and substantial heterogeneity, these findings should be interpreted cautiously.

## Data Availability

The original contributions presented in the study are included in the article/Supplementary material, further inquiries can be directed to the corresponding author.
