# Supplementary material for: A systematic review and meta-analysis of high-intensity interval training for mental health and executive function in university students
Source: Front Psychol. 2026 Jul 24;17:1891095. doi: 10.3389/fpsyg.2026.1891095 (PMC13447482; doi:10.3389/fpsyg.2026.1891095)
Supplement: Supplementary file 1 [file Supplementary_file_1.DOCX]

**Table S1. Search Strategy**

| **Database** | **Search Query** | **Number of records** | | | |
| --- | --- | --- | --- | --- | --- |
|  |  | **Initial search** | **Updated search** | **Total** |  |
| WOS | TS=(("high intensity interval training" OR "high-intensity interval trainings" OR "high intensity intermittent training" OR "high intensity interval exercise" OR "high intensity intermittent exercise" OR "high intensity circuit training" OR "high intensity training" OR "high intensity exercise" OR "high intensity aerobic interval training" OR "sprint interval training") AND (cognit* OR perceptual OR attention OR memory OR executive OR psycholog* OR mental OR self-concept OR self-esteem OR self-worth OR satisfaction OR anxiety OR mood OR affect OR enjoyment OR compliance OR adherence OR resilienc* OR depress* OR stress OR pressure) AND (university OR college)) | 326 | 55 | 381 |  |
| Scopus | ( TITLE-ABS-KEY ( ( "high intensity interval training" OR "high-intensity interval trainings" OR "high intensity intermittent training" OR "high intensity interval exercise" OR "high intensity intermittent exercise" OR "high intensity circuit training" OR "high intensity training" OR "high intensity exercise" OR "high intensity aerobic interval training" OR "sprint interval training" ) ) AND TITLE-ABS-KEY ( ( cognit* OR perceptual OR attention OR memory OR executive OR psycholog* OR mental OR self-concept OR self-esteem OR self-worth OR satisfaction OR anxiety OR mood OR affect OR enjoyment OR compliance OR adherence OR resilienc* OR depress* OR stress OR pressure ) ) AND TITLE-ABS-KEY ( ( university OR college ) ) ) | 331 | 85 | 416 |  |
| Pubmed | ((("high intensity interval training"[Title/Abstract] OR "high-intensity interval trainings"[Title/Abstract] OR "high intensity intermittent training"[Title/Abstract] OR "high intensity interval exercise"[Title/Abstract] OR "high intensity intermittent exercise"[Title/Abstract] OR "high intensity circuit training"[Title/Abstract] OR "high intensity training"[Title/Abstract] OR "high intensity exercise"[Title/Abstract] OR "high intensity aerobic interval training"[Title/Abstract] OR "sprint interval training"[Title/Abstract])) AND ((cognit*[Title/Abstract] OR perceptual[Title/Abstract] OR attention[Title/Abstract] OR memory[Title/Abstract] OR executive[Title/Abstract] OR psycholog*[Title/Abstract] OR mental[Title/Abstract] OR self-concept[Title/Abstract] OR self-esteem[Title/Abstract] OR self-worth[Title/Abstract] OR satisfaction[Title/Abstract] OR anxiety[Title/Abstract] OR mood[Title/Abstract] OR affect[Title/Abstract] OR enjoyment[Title/Abstract] OR compliance[Title/Abstract] OR adherence[Title/Abstract] OR resilienc*[Title/Abstract] OR depress*[Title/Abstract] OR stress[Title/Abstract] OR pressure[Title/Abstract]))) AND ((university[Title/Abstract] OR college[Title/Abstract])) | 141 | 41 | 182 |  |
| SportDiscus | AB (("high intensity interval training" OR "high-intensity interval trainings" OR "high intensity intermittent training" OR "high intensity interval exercise" OR "high intensity intermittent exercise" OR "high intensity circuit training" OR "high intensity training" OR "high intensity exercise" OR "high intensity aerobic interval training" OR "sprint interval training")) AND AB ((cognit* OR perceptual OR attention OR memory OR executive OR psycholog* OR mental OR self-concept OR self-esteem OR self-worth OR satisfaction OR anxiety OR mood OR affect OR enjoyment OR compliance OR adherence OR resilienc* OR depress* OR stress OR pressure)) AND AB ((university OR college)) | 72 | 15 | 87 |  |
| MEDLINE | AB (("high intensity interval training" OR "high-intensity interval trainings" OR "high intensity intermittent training" OR "high intensity interval exercise" OR "high intensity intermittent exercise" OR "high intensity circuit training" OR "high intensity training" OR "high intensity exercise" OR "high intensity aerobic interval training" OR "sprint interval training")) AND AB ((cognit* OR perceptual OR attention OR memory OR executive OR psycholog* OR mental OR self-concept OR self-esteem OR self-worth OR satisfaction OR anxiety OR mood OR affect OR enjoyment OR compliance OR adherence OR resilienc* OR depress* OR stress OR pressure)) AND AB ((university OR college)) | 150 | 76 | 226 |  |

Note: The initial search covered database inception to December 2024, and the updated search was conducted in April 2026.

**Table S2. Leave-one-study-out sensitivity analysis**

| **Omitted Study** | **Estimate** | **CI Lower Bound** | **CI Upper Bound** | **P-value** |
| --- | --- | --- | --- | --- |
| **Mental ill-health** |  |  |  |  |
| Yin (2024) | -0.7673677 | -1.3221042 | -0.2126312 | 0.0067036 |
| Philippot (2022) | -0.7506501 | -1.3134136 | -0.1878866 | 0.0089404 |
| Wang Yu (2023) | -0.8356462 | -1.3453226 | -0.3259698 | 0.0013114 |
| Yalman (2021) | -0.7469460 | -1.3061161 | -0.1877759 | 0.0088410 |
| Eather (2019) | -0.7937531 | -1.3558631 | -0.2316431 | 0.0056461 |
| Lucibello (2020) | -0.8010056 | -1.3445934 | -0.2574179 | 0.0038756 |
| Guo (2023) | -0.6011712 | -1.0693358 | -0.1330066 | 0.0118428 |
| Chang (2024) | -0.7176026 | -1.2839856 | -0.1512195 | 0.0130187 |
| Zhang (2021) | -0.7969082 | -1.3423376 | -0.2514787 | 0.0041881 |
| Fu (2025) | -0.7322655 | -1.2945180 | -0.1700131 | 0.0106918 |
| Thenmozhi (2025) | -0.5287824 | -0.8958991 | -0.1616656 | 0.0047567 |
| **Executive function** |  |  |  |  |
| Wang Xiaomei (2023) | 0.4577758 | -0.0779240 | 0.9934756 | 0.0939610 |
| Eather (2019) | 0.4364732 | -0.0710440 | 0.9439903 | 0.0918726 |
| Guo (2023) | 0.2704177 | -0.1056948 | 0.6465302 | 0.1587830 |
| Wallenwein (2025) | 0.5490723 | 0.1603601 | 0.9377845 | 0.0056310 |
| Jiménez-Roldán (2025) | 0.2882431 | -0.1698926 | 0.7463788 | 0.2175231 |

**Figure S1. Forest plot of subgroup analysis of mental ill-health**

**Figure S2. Forest plot of subgroup analysis of executive function**
